# When Do Short-Wave Cones Signal Blue or Red? A Solution Introducing the Concept of Primary and Secondary Cone Outputs

**DOI:** 10.1371/journal.pone.0154048

**Published:** 2016-04-22

**Authors:** Ralph W. Pridmore

**Affiliations:** Central Houses Pty Ltd, 8c Rothwell Rd, Turramurra, Sydney, NSW, 2074, Australia; University of Melbourne, AUSTRALIA

## Abstract

A recent paper by Oh and Sakata investigates the “incompletely solved mystery” of how the three cone responses map onto perceived hue, and particularly the S cone’s well-known problematic contribution to blueness and redness. Citing previous workers, they argue the twentieth century traditional multistage model does not satisfactorily account for color appearance. In their experiment, increasing S cone excitation with shortening wavelength from about 480–460 nm increased perceived blueness up to the unique Blue point at 470 nm, when (a) it began decreasing and (b) redness perception began increasing. The authors asked, What mechanism can be responsible for such functions? I demonstrate a solution. First, it is shown the problem does not lie in the traditional opponent color chromatic responses yellow-blue, red-green (*y-b*, *r-g*, which accurately predict the above functions), but in the traditional multistage model of mapping cone responses to chromatic response functions. Arguably, this is due to the S cone’s hypothetically signaling both blueness and redness by the same mechanism rather than by different, independent, mechanisms. Hence a new distinction or mechanism is proposed for a more accurate model, that introduces the new terms primary and secondary cone outputs. However, this distinction requires that the cones S, M, L each directly produce one of the three spectral chromatic responses *b*, *g*, *y*. Such a model was recently published, based on extremely high correlation of SML cone responsivities with the three spectral (*bgy*) chromatic responses. This model encodes the former directly onto the latter one-to-one as cone primary outputs, whilst S and L cones have a further or secondary function where each produces one of the two spectral lobes of *r* chromatic response. The proposed distinction between primary and secondary cone outputs is a new concept and useful tool in detailing cone outputs to chromatic channels, and provides a solution to the above “incompletely solved mystery.” Thus the S cone has a primary output producing the total *b* chromatic response and a secondary output that shares with the L cone the production of *r* chromatic response, thus aligning with Oh and Sokata’s results. The model similarly maps L cone to yellowness as primary output and to redness as secondary output.

## Introduction

A recent paper [1] by Oh and Sakata investigates the “incompletely solved mystery” of how the three classes of cones map onto percepts of hue, and particularly the short wave (S) cone’s contribution to hue via the opponent color channels. They note previous researchers have found the S cone’s contribution to blueness is problematic, and that the four unique hues do not align in any simple manner with various theoretical axes for opponent or single chromatic channels in various chromaticity diagrams,[2–5] and conclude that the traditional mid-twentieth century stage model does not satisfactorily account for color appearance. They found the traditional equivalence between cone-opponency (eg, L-M) and hue-opponency (eg, red-green) no longer seemed assured but questionable. That is, the phenomenal blue-yellow relationship, and its line in chromaticity space, does not depend solely on modulation of S cone signals. Researchers had shown that perceived blueness does not depend monotonically on the level of S cone excitation but also on the relative excitation of M and L cones[6], as implied by the curved loci of unique and other constant hues in color space[7,8]. The S cone is traditionally held to contribute to blueness and also to short-wavelength redness[9,10], or alternatively to redness by inhibiting M cone signals[11]. Drum [12] however argues that his and other data show that S cone activity produces a reddish purple hue at short wavelengths and (indirectly) a yellow hue at middle and long wavelengths. Furthermore, Ingling [10] has claimed that S cone contributions to the *r-g* channel are (counter-intuitively) greater for desaturated signals, and spatial frequency decides whether the signals are chromatic or achromatic. In general, more problematic complexities in cone contributions to hue are reported for the ancient S cone than other cones.

Oh and Sakata’s recent experiments (detailed below) further explored the degree of monotonicity in the contribution of S cones to chromatic channels. They found that increasing the S cone excitation stimulates a non-monotonic function in perceived blueness. The change in function occurs near the wavelength of unique blue, which also marks the commencement of redness perception. They ask, What mechanism could be responsible for this pattern? This paper proposes a solution below, which may also be taken to answer the general question (this article’s title), When do S cones signal blue or red?

**Fig 1:**
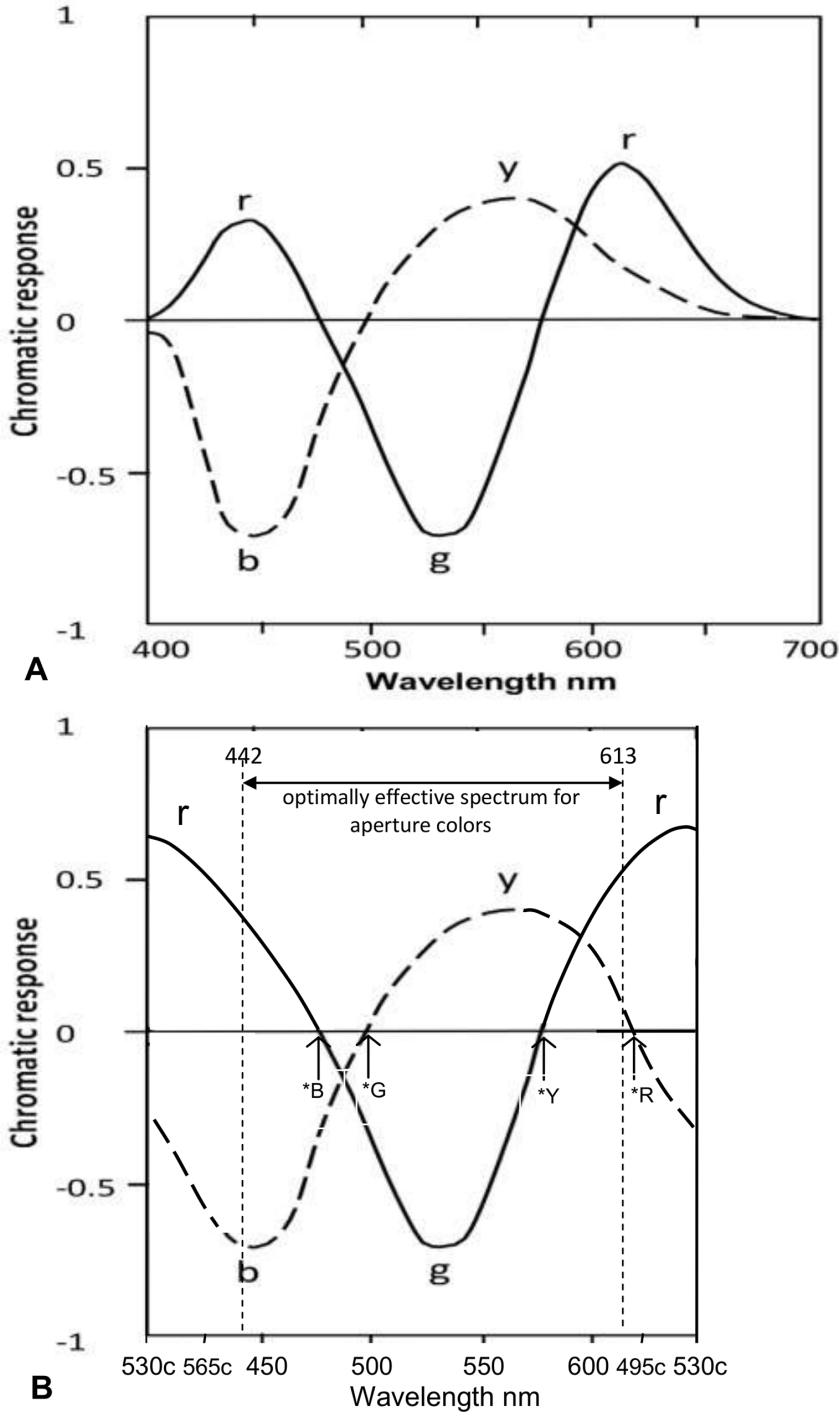
(A). Opponent color chromatic responses *y-b*, *r-g*, over the spectrum, adapted from Reference 9. This shows *r* response as two spectral peaks about 440 and 610 nm. (B). Same as Fig (A) but with chromatic responses shown over the whole hue cycle, with nonspectral purples (beyond the effective spectrum extremes at 442 and 613 nm, wavelength limits to monochromatic optimal color stimuli [16]) given an arbitrary interval equivalent to some 70 nm on x-axis. This shows the *r* curve peak (colorimetrically calculated from the indicated two spectral *r* peaks of relative amounts 0.67:1 ratio) about 510 c, with unique Red (labeled *R) about 494 c from previous data. The nonspectral purples are indicated by their complementary wavelengths, e.g, 510 c.

It is first appropriate to summarize the traditional transformations from cone sensitivities to opponent color chromatic responses. Fig 1 shows Hurvich and Jameson’s opponent color chromatic responses[9,13–15], based on experimental hue cancellation. Such traditional stage models derive blueness mostly or entirely from the S cone and similarly greenness mostly or entirely from the medium-wave (M) cone, since the *b* and *g c*hromatic response peaks are very similar to the S and M cone response peaks (445 and 535 nm approx). Equations below are from Ref. 15, perhaps the simplest of traditional models in encoding S and M cones directly to *b* and *g*:

y−b=(0.15M+0.15L−1.0S) Eq 1

r−g=(0.55S+0.55L−1.0M) Eq 2

The other two unique hues derive from pairs of cones: redness from the S and long wave (L) cones, and yellowness from M and L cones in roughly equal parts. The latter is problematic since the *y* chromatic response peak and L cone response peak are nowadays known to be very similar wavelengths (approx 560–570 nm), suggesting direct correlation, but traditional models have always derived *y* from M + L cones, possibly because the previous terms for the M and L cones were G and R, and because hues G + R produce Y.

An important part of the traditional model is the role played by S and L cones in producing the two spectral *r* lobes of redness chromatic response in Fig 1A. The two *r* lobes admix to form the *r* chromatic response that spans the entire nonspectral part of the perceived hue cycle. It is noteworthy that both lobes represent redness *r* (according to hue cancellation experiments [9]), although one is produced from the S cone and one from the L cone. How the S cone, that normally stimulates blueness, can produce a lobe representing redness *r* has never been explained physiologically. But the same ability applies when the S cone contributes simultaneously to both blue and red in perceived violet (short-wave) colors.

Fig 1A shows the graph over the spectrum, with two spectral *r* lobes. But this omits the nonspectral area, where *all* colors are perceived as containing redness and where unique Red lies (about 494 c) and where redness peaks (about 510 c, calculated from the relative amplitudes–approximate ratio of 0.67:1.0—of the two *r* lobes in Refs. 9 and 14). To this end, Fig 1B allows an arbitrary interval [16] on the x-axis for the nonspectral or purple region of the hue cycle in order to represent the *r* chromatic response over the total hue cycle. The arrangement of the four hues in Fig 1B is in agreement with hue prediction [13] and hue naming data [17], and shows perceived redness starts at unique Blue and increases amplitude with shortening wavelength in the short-wave end of the spectrum (in exact agreement with Oh and Sakata’s results, below).

### Oh and Sakata’s Experiment

Oh and Sakata’s experiment [1] measured S cone excitation versus perceived hue strength. Their results show increasing excitation of S cones, relative to constant excitation of L and M cones, contributes to perceived blueness and redness. A series of S cone isolating stimuli were tested. The 10 chromatic stimuli (on CRT monitor) lay on a tritanopic confusion line in CIE 1931 chromaticity diagram (where normal trichromats but not tritanopes see blueish colors), with high saturation and dominant wavelength varying from 458 to 477 nm.

**Fig 2:**
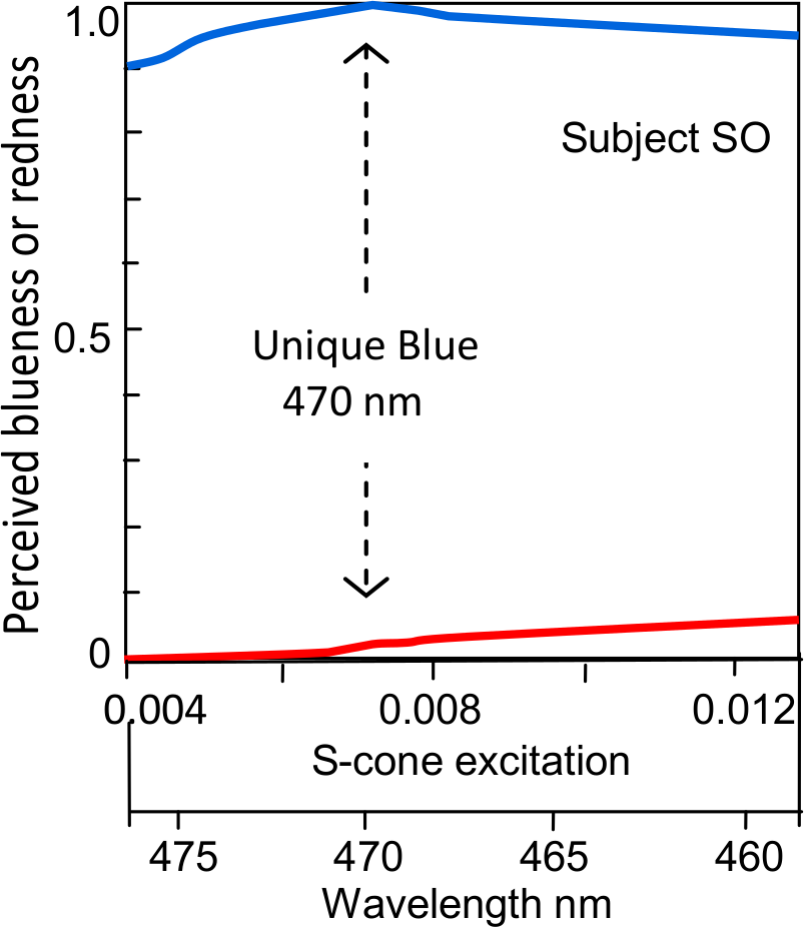
Test results for SO, one subject of 5 who were tested for both blueness (blue line) and redness (red line), graphed from Ref. 1 data. The x-axis shows S cone excitation and dominant wavelength digitized from Fig 1 of Ref. 1 and CIE table of chromaticity coordinates for wavelengths. Peak blueness occurs near unique Blue (470 nm), where redness first commences.

Fig 2 shows the combined experimental results for one subject SO, approximating the average for all subjects (8 subjects for the blueness test, and 5 for the redness test). The x-axis shows wavelength and S cone excitation; the latter increases with decreasing dominant wavelength. Perceived hue changes from greenish blue at about 480 nm to pure blue about 470 nm and to reddish blue as wavelength decreases to about 460 nm. Redness commences at 470 nm and increases with shortening wavelength. The authors appropriately ask, “What mechanism could be responsible for this pattern of correspondence between S-cone increment and perceived hue?” This paper proposes a solution based on previous experimental data.

## Proposed Solution

The solution comprises two parts: (1) the well known hue prediction method [13] from traditional opponent color chromatic responses, and (2) a recently published schema [18] of cone outputs to opponent color chromatic responses, whose output schema is rather simpler than the traditional schema. The solution’s first part lies in the last or second stage (color appearance) of multistage theories of color vision [14,15] but is treated first as it is considerably simpler. The solution’s second part concerns the first stage (retinal receptor layer) of multistage theory.

**Fig 3:**
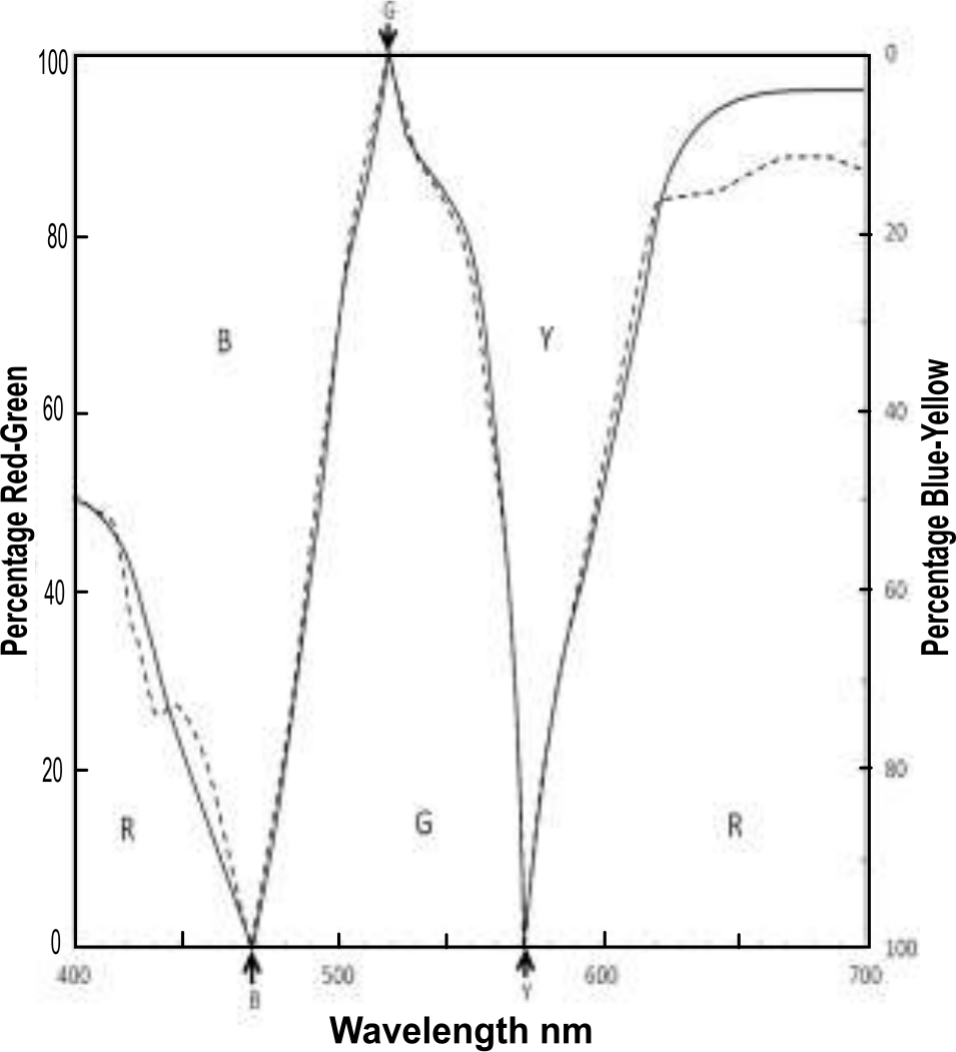
Hue naming and hue prediction data from Werner & Wooten 1979 [17] with permission. Black solid lines: Hue naming data for 3 subjects (mean). Black dotted lines: Hue prediction for same 3 subjects using Hurvich & Jameson model [13]. Arrows indicate unique hue loci at 100% R or G (left ordinate), and B or Y (right ordinate).

The first part of the solution is a traditional mechanism apparently overlooked by Oh and Sakata but detailed herewith (as a worked example) to demonstrate its effectiveness. The results in Fig 2 show that perceived blueness rises to a peak about unique Blue (470 nm is the mean for 8 subjects) near the point where redness commences and then increases with decreasing wavelength. These hues, as the authors note, vary from reddish blue through unique Blue to greenish blue. This change of hues with wavelength is a well known phenomenon and is no mystery. It is satisfactorily predicted by the arrangement of traditional chromatic responses in Fig 1, particularly if formulated as Hurvich and Jameson’s hue coefficients [13], which predict the variations of hue and hue strength shown in Fig 3. This result was later confirmed by Werner and Wooten’s hue naming experiment [17], also shown in Fig 3. The two methods’ agreement (using the same three subjects) supports their predictive accuracy, and in addition, implies that each chromatic response curve represents one pure or unique hue (as described later below).

Given that the model depicted in Fig 3 satisfactorily predicts the perceived hue results in Fig 2, it seems this part (opponent chromatic responses) of the traditional multistage model is not at fault. So the above-mentioned problem^1^ with the traditional stage model instead appears to lie in the traditional “wiring diagram” or schema encoding cone outputs to chromatic responses. This hypothetical mechanism linking first and second stages of color vision is contentious and its complexity (according to reliability theory [19]) renders it more likely to have potential errors. To properly address this area, and in view of Ref. 1’s noting other problems between cone sensitivities’ transformation to phenomenological hue, the present article considers not only the S cone but other cones’ contributions to perceived hue.

**Fig 4:**
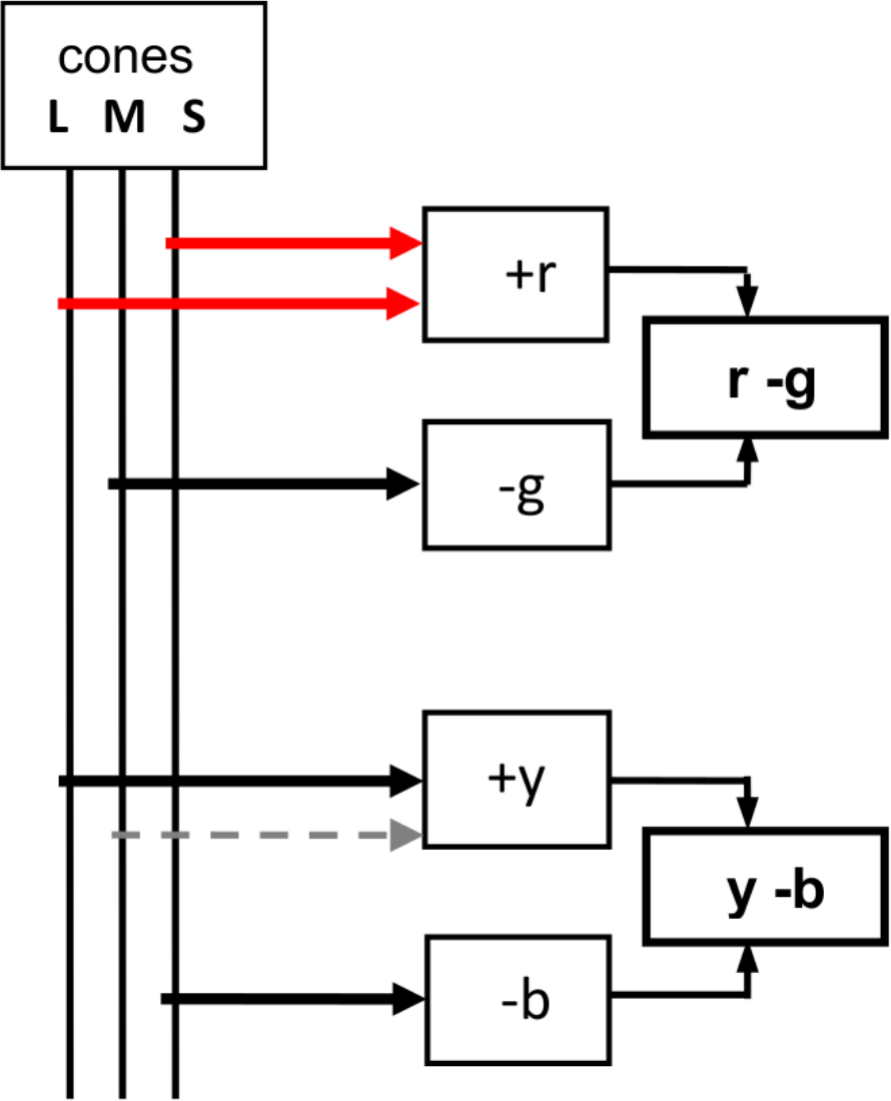
Traditional schema or wiring diagram encoding SML cone outputs to opponent color chromatic responses *r—g*, *y—b*. Removing dashed grey line (contentious M input to *y*) transforms the schema to the simpler more symmetrical model in Ref. 18, wh+ere S = *b*, M = *g*, L = *y* and, as in tradition, S + L = *r*. In this version of wiring diagram [18] (with grey line omitted), the horizontal black links from SML cones to *bgy* chromatic responses represent cone *primary* outputs, and the two red lines represent cone *secondary* outputs where two cones S and L combine to form the redness chromatic response.

The traditional opponent color model or schema of cone outputs to opponent chromatic responses is shown in Fig 4. (Note the dashed grey line is part of the traditional diagram, and later below is omitted to represent a more recent and simpler wiring diagram.) It can be seen that the S cone contributes (totally) to *b* and (partly) to *r* so this link is closely related to our problem of understanding the S cone’s contribution to blueness and redness. However, its part in the overall schema is difficult to assess as the schema lacks simplicity and is possibly erroneous. An example is the L cone output. One might expect S and L cones to have a similar pattern of contributions to the chromatic responses because S and L cones are closely related in two ways. First, S and L are the original cones in early primate dichromatic color vision [20]. The cone peaks are complementary wavelengths so they are clearly a related pair; logic implies the pair were, and remain, complementary in order to separately produce different hues—blue and yellow—yet in combination admix white so sunlight/daylight is perceived as white rather than colored. Second, S and L cones together form the *r* chromatic response. (M or its product, *g* chromatic response, cannot contribute to *r* as it opposes *r* chromatic response.) But in contrast, L cone output in the traditional schema is not of a parallel or similar type to S cone’s output but quite different: it contributes partly to each of two chromatic responses, *y* and *r*. The most recent cone, M, contributes the total *g* chromatic response and about half the *y* response (see Eq 1), and so it supposedly contributes more to chromatic responses than does the older L cone. This schema seems an unduly complicated and asymmetrical distribution of cone outputs. In such a long-evolved system as trichromatic color vision, one expects simple and symmetrical mechanisms (though admittedly color science is taking a long time to find them).

However, an alternative and simpler schema was recently published [18] as a byproduct of measuring correlation between SML cone responsivities and spectral (*bgy*) chromatic responses. The extremely high correlation coefficients indicate each SML cone outputs directly to one spectral (*b*, *g*, or *y*) chromatic response, with S and L having a further function in directing secondary outputs towards forming *r* chromatic response. The two types of output (later below termed primary and secondary) offer a means of further delineating cone outputs to particular chromatic responses. Here is a symmetrical mechanism that promises to simplify the problems identified in Ref. 1, including the S cone’s contributions to hue. Its basis in experimental data, as detailed in Ref. 18, is therefore described briefly below.

But first it is worth noting that Ref. 18 made another important deduction concerning the chromatic responses, which affects this paper’s ability to predict which cones signal which hues. It was noted above (in the second paragraph of this section “Proposed Solution”) that the two methods [13,17] of hue naming closely agreed, as shown in Fig 3. The success of the two methods, and their agreement, imply that each chromatic response curve (eg, the *b* response, described by the experimenters [17] as representing “blueness”) represents a pure hue over its total wavelength range. The alternative possibility is that each chromatic response curve (e.g., *b*) represents a variety of bluish hues between cyan and violet. This however is not supported by the success of the two methods (and their math formulas [18]) in hue naming, which necessarily requires that each chromatic response curve *b*, *g*, *y*, or *r*, represents one single quality over all its wavelength range. The formulas with their math terms *b*, *g*, *y*, and *r*, would not work unless each of the terms were a single invariant quality or hue. That hue can only be perceived at the equilibrium wavelength, which represents the unique hue, as the remainder of the response curve is overlaid by other response curve(s). Hence Ref. 18 deduces that each chromatic response curve represents a unique hue over all its wavelength range. This is an important and far-reaching deduction, and has been agreed by Professor Jack Werner, one of the original experimenters[17], as noted in Ref. 18 (in Reader’s Comment posted by Pridmore). This therefore clarifies which hue is signaled by which cone and/or chromatic response.

**Fig 5:**
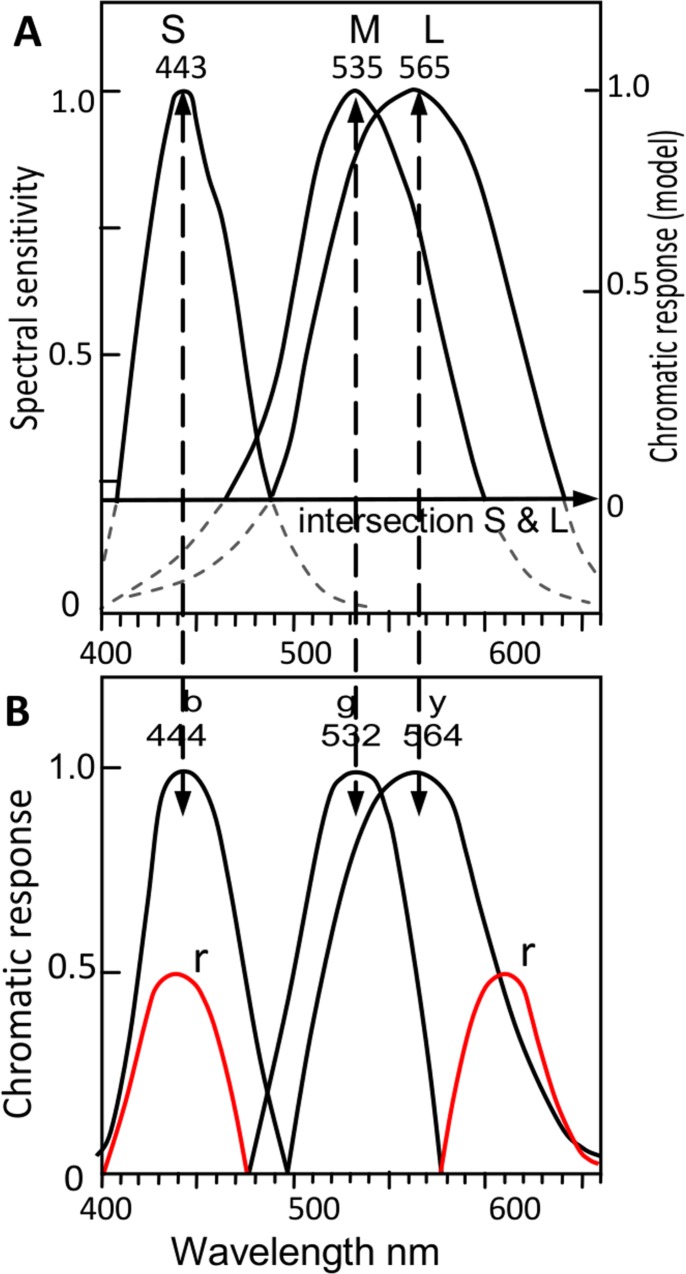
Black curves: Cone and chromatic response curves from the cited sources, normalized at 1.0 response and with wavelength peaks aligned with Table 1 means. Vertical dashed arrowed lines show identical wavelengths ± 2 or 3 nm for curve peaks in Fig (A) and curve peaks in Fig (B). (**A).** Black curves: Cone sensitivity estimates of Stockman & Sharpe [34]. Arrowed horizontal line from S and L curves’ intersection [equivalent to intersection of *b* and *y* chromatic response curves in Fig (B)] indicates null response of new chromatic response model at right *y*-axis. (This interval 0–1 is equal to 0–1 interval in Fig (B) below, allowing comparison of curves to the same shared scale). Cone sensitivities below intersection of S and L curves (arrowed line) are shown in gray, and are discounted from comparison. (**B)**. Red lines: Spectral (*bgy*) opponent chromatic response curves from Hurvich in Fig 1A. Red curves: Spectral lobes for *r* chromatic response normalised at 0.5 response.

The extensive data in Table 1 [12,17,21–34] (from Ref. 18) show the mean wavelength peaks of SML cone responses (physiologically measured) are identical to those of *bgy* chromatic responses (psychophysically measured) ± 3 nm; that is, the means are 444, 535, 565 ± 2 nm. Ref. 18 calculates the correlation coefficient between SML and *bgy* wavelength peaks in Table 1 as extremely high at 0.9998. However, comparison of curves also requires comparison of curve math functions besides wavelength peaks. Accordingly Fig 5 illustrates the SML curves and the individual *bgy* curves plotted linearly to the same graph axes for response and wavelength, to allow fair comparison. These curves are usually represented in the literature in mixed terms, with SML curves in log and/or wavenumber and *bgy* curves in linear and in opposed signs, making comparison difficult-to-impossible. The normalised SML cone responses (Fig 5A) and the normalised chromatic responses *bgy* (Fig 5B) are aligned with their mean wavelength peaks from Table 1 for the spectral hues. (Also shown in Fig 5B, in red, are the two spectral *r* curves normalised at only 0.5 as they combine to form redness *r*; the following few paragraphs concern mainly the spectral chromatic responses.)

**Table 1:** Wavelength peaks of human cone spectral sensitivities and of opponent chromatic responses from hue cancellation experiments (from Pridmore [18]).

Cone sensitivity peaks from psychophysical				Opponent-color chromatic response peaks from hue				
and experimental data				cancellation experiments				
*S*	*M*	*L*	References	*b*	*g*	*y*	*r*	References
440	540	565	Smith [25]	435	530	550	440, 620	Jameson [9]
445	540	560	Judd [26]	440	530	555	440, 610	Romeskie [21]
444	527	571	Estevez [27]	455	525	582	443, 610	Werner [17]
450	540	560	Wyszecki [28]	447	535	565	440, 610	Takahashi [22]#
440	540	565	Wyszecki [29]	445	530	565	445, 615	Takahashi [22]†
444	530	571	Wyszecki [30]	445	530	564	440, 610	Takahashi [22]*
438	533	564	Dowling [31]	445	535	570	442, 610	Takahashi [22]#L
445	535	570	Vos [32]	445	535	565	442, 610	Takahashi [22]†L
440	530	560	Stockman [33]	440	540	560	460, 610	Fuld [23]
440	540	565	Stockman [34]	440	525	560	440, 610	Kulp [24]
*443*	*535*	*565*	*Means*	*444*	*532*	*564*	*443*, *612*	*Means*

These data represent, or are calculated from, experimental data and represent all or most of the data sets available. Under “References” only first authors are listed. Wyszecki [28], [29], and [30], refer respectively to Wyszecki & Stiles (1967) Konig-type fundamentals, Vos & Walraven (1978) fundamentals, and Stiles (1953, 1959) field sensitivities for π_1,_ π_4,_ and π_5_ mechanisms. In Fuld (1991), and in Kulp & Fuld (1995), data are given at large (20 nm) intervals so mean data for the 3 or 4 subjects respectively were plotted, curves drawn and peaks interpolated to nearest 5 nm as listed below. Takahashi’s very thorough study [11] gives several troland levels for 8700 K color temperature and 2 subjects:

# denotes 50 td

† is 500 td, and

* is 5000 td.

Two troland levels are shown for 5200 K and 1 subject:

#L denotes 50 td

†L is 500 td.

**Fig 6:**
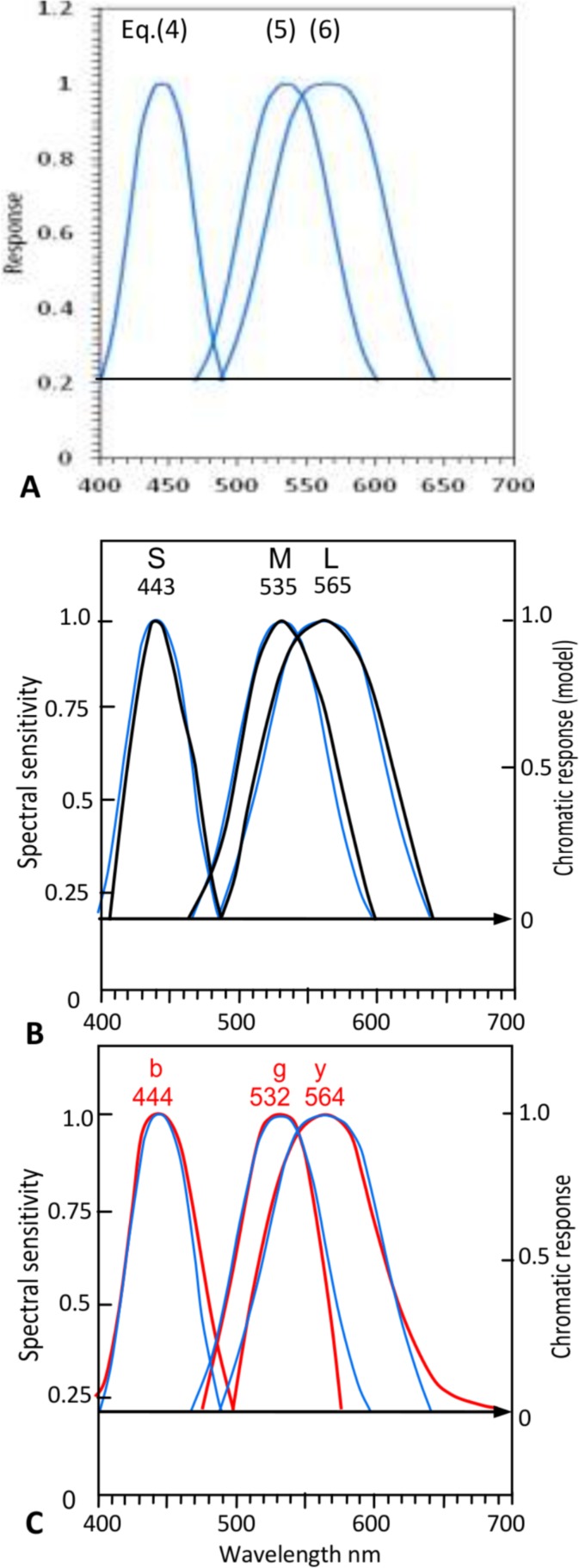
Curve fitting prediction of cone sensitivities and unique hue chromatic responses. **A**. Three formulaic curves in blue [plotted from Eqns (4)-(6) of Ref. 18] predicting the SML cone curves and the *bgy* chromatic response curves. Their wavelength peaks are nominally 445, 535, 565 nm but can be shifted horizontally to any wavelength without changing curve shape. **B**. The formulaic curves (in blue) compared to SML cone response curves in black (from Fig 5 above) by overlapping the former’s wavelength peaks with the latter as labeled. **C.** The same formulaic curves compared to *bgy* chromatic response curves in red (from Fig 5) by overlapping the former’s wavelength peaks with the latter as labeled. The predicted curves (in blue) correlate very closely with cone response curves to a mean 0.987 correlation coefficient, and with chromatic response curves to a mean 0.967 correlation coefficient [18].

Note in Fig 5B that the intersection of *b* and *y* chromatic responses at zero serves traditionally as the zero response level for all chromatic responses. Consequently the cone sensitivity curves (whose slopes become almost horizontal as they approach the spectrum extremes) are cut off below the intersection of the S and L cone curves (which correlate to the *b* and *y* curves), to serve as their zero response level in accord with that of the chromatic responses. This now enables cone curves and chromatic response curves to be compared all in the same scale in Fig 5. The curve shapes are notably similar, with S and *b* the narrowest, M and *g* rather wider, and L and *y* the widest. A quantitative correlation of the two curve sets may be made by comparing the curves’ math functions, or showing that one set of math functions [18] suffices to predict both sets of curves, as is shown in Fig 6. The three math functions [18] (in blue) predicting the cone curves correlate with the SML curves at an average coefficient of 0.987, and with the *bgy* chromatic response curves at an average 0.967. Hence, for practical purposes, the SML curve set and the *bgy* curve set are equivalent sets, implying a direct one-to-one relationship between the two sets. (Ref. 18 goes on to closely correlate yet another parameter of the two sets.)

The extremely high correlation between SML cones and the spectral chromatic responses *bgy*, combined with deterministic causation of the latter by the former as detailed in Ref. 18, implies that SML cone outputs lead directly to the postreceptoral *bgy* chromatic responses (and therefore the spectral unique hues blue, green, yellow). Although such simple relationships in color vision may be unusual, there is no reasonable alternative relationship in this case. This supports the traditional multistage models in the case of *b* and *g* chromatic responses (in their allocation of all or most of S cone to *b* chromatic response and of all or most of M cone response to *g* chromatic response) but not the *y* response derived in tradition from both M and L cones. The Ref. 18 schema in algebraic terms is as follows (with approximate proportions for *r*):

y−b=(1.0L−1.0S) Eq 3

r−g=(0.4S+0.6L−1.0M) Eq 4

This is shown diagramatically in Fig 4, if the grey line (linking M and *y*) is omitted. This simple change represents the major difference between the new paradigm [18] and the traditional or standard multistage model. The single change transforms the mechanism into a simple and symmetrical distribution mechanism, as illustrated more fully in Fig 5.

This schema (Figs 4 or 5) also provides a simpler explanation for the origin of unique hues than other attempts (see Ref. 18 for a list of references), and hence the principle of parsimony (also known as Occams’ Razor) may be applied in its support.

Now, the revised schema of transformations in Fig 4 and in Fig 5, where each cone S, M, or L directly and totally produces a spectral hue *b*, *g*, or *y* (without interference or contribution from any other cone), allows a new concept: That is, each cone has a primary function or output (curves in black) and may also have a secondary function (curves in red). Fig 5 shows that all three cones have a *primary function* or output, in each producing one entire spectral chromatic response curve *b*, *g*, or *y*. Dashed vertical arrowed lines indicate the almost-identical peak wavelengths for SML and *bgy*. In addition, two cones S and L have a further or *secondary function*, in producing the two spectral *r* lobes near 442 and 613 nm, together producing by admixture the (largely nonspectral) *r* chromatic response. The primary outputs of the three cones are shown as chromatic responses in black and the secondary outputs as chromatic responses in red. This concept is not possible in the traditional transformation schema(s) [35] where two cones may contribute to the same spectral hue *b*,*g* or *y*.

The schema in Ref 18, in combination with Hurvich and Jameson’s well known hue prediction method [13], provides a mechanism accounting for Fig 2 experimental results concerning the S cone. That is, increasing S cone excitation varies perceived blueness (in exact accord with traditional opponent color chromatic responses, see Figs 1 and 3) as a *primary function*, and varies perceived redness as an independent *secondary function*. Similarly, the common observation that the L cone influences both yellowness and redness continues to be true but with the distinction that *y* is a primary function and *r* is a secondary function of the L cone outputs.

The bases of the primary and secondary hierarchies in cone output are different. The basis of the *primary* output is the SML cone fundamental spectral responsivities, producing postreceptorally and directly the *bgy* chromatic responses (whose perceived hues, as already mentioned, are unique Blue, Green, Yellow [18]). The basis of the *secondary* output is the two spectral lobes about 442 and 613 nm (evident in hue-cancellation experiments). These are apparently output from S and L cones separately from their fundamental responsivities, and produce postreceptorally the *r* (unique Red) chromatic response function which is largely nonspectral and is thus produced by neural admixture of the two spectral *r* lobes. See Figs 5, 1A and 1B. The lobes’ wavelength peaks are supported by the wavelengths of the optimal compound (i.e., nonspectral) color stimuli for aperture colors, comprising varying amounts of only two wavelengths 442 and 613 nm ±1 in all CIE standard illuminants [36,37].

### Further Considerations

Given the proposal of cone secondary outputs, what is their difference from primary outputs? The secondary outputs are well differentiated from the primary outputs in either sign or wavelength. Consider the S cone’s primary output (to *b* chromatic response), which by convention is a negative response. In contrast, the S cone’s secondary output (to *r* chromatic response) is a positive response–see Fig 1A or Fig 4. Next consider the L cone’s primary output (to *y* chromatic response), whose wavelength peak is about 565 nm; in clear contrast, the wavelength peak of the secondary output (to the long wavelength lobe of *r* chromatic response) is about 613 nm.

More information on the new concept of cone secondary outputs is required. For instance, What is the difference between them and primary outputs, and what physiological evidence supports their existence? In the first case, it can be noted that cone primary outputs concern the spectral (*bgy*) unique hues only, and that each cone primary output represents the total contribution to its corresponding chromatic response. In contrast, cone secondary outputs concern only the *r* chromatic response (which is largely nonspectral) and its two spectral lobes near 442 and 613 nm; and in no case does a cone secondary output represent the total contribution to any chromatic response, but only a partial contribution. Namely, it contributes one of the two spectral lobes to *r* chromatic response.

There is no known physiological difference between the primary and secondary outputs in the literature, since the concept of the difference is only recent. The literature, to my knowledge, reveals only one possible piece of physiological support. One might expect a secondary peak of the cone response curve to represent a secondary output but the only possible sign of such is the pronounced secondary peak in the long wavelength region of the Wyszecki and Stiles bleaching model and alternative bleaching model of the S cone.^38^ For both models, the S cone response peaks near wavenumber 22.5 cm^-1^ (or 445 nm) and the secondary peak occurs near wavenumber 16.4 cm^-1^ (or 610 nm). The latter is the same peak as the long wavelength lobe of the *r* response in hue cancellation data. However, these bleaching models are questionable versions of the S cone response (also known as “blue” cone pigment fundamental) since they differ considerably from other researchers’ models [38], which do not show a secondary peak. Furthermore, the secondary peak would infer that the S cone not only provides the primary input to *b* chromatic response but the secondary inputs to both the short wavelength and long wavelength lobes of *r* chromatic response. That problematically omits the L cone secondary contribution to *r* chromatic response. In sum, this possibility is intriguing but not convincing.

The question is sometimes asked, why do very short wavelengths stimulate red? What is the advantage to the visual ecology? Two reasons appear, and both relate to the continuity of changing hue across the nonspectral gap between the spectrum extremes, i.e., across the nonspectral purples in the psychophysical hue cycle (e.g., as shown in the x-axis of Fig 1B). First, the long wavelength spectrum extreme is perceived as reddish (yellowish red with increasing redness with longer wavelength), and the short wavelength spectrum extreme is also perceived as reddish (reddish blue, also with increasing redness as the wavelength extreme is approached), because these hues are quite near each other, to either side of the nonspectral purples. Hence, when we see a (blue-and-red) purple we correctly expect it to lie between violet and red in the psychophysical hue cycle, and hence the average or naïve observer is not aware of the physical spectrum or that the purples are nonspectral. The redness of each spectrum extreme (in the lab or in the perceived rainbow) indicates the nearness of these reddish hues, and that the in-between hues are even redder. Second, given that unique blue is located short of the spectrum extreme, and given that the longer wavelength side of unique blue appears greenish, then the other (shorter wavelength) side must appear some other hue, either red or yellow as the two remaining unique hues. It must be red since yellow is complementary to blue and therefore cannot be adjacent, or its mixture with blue will not be chromatic but increasingly achromatic.

A side effect of cataract operations is sometimes that a unique hue has to be relearnt, as some colors now (post-operation) appear slightly different hues. Does this fact affect the credibility of such a simple relationship between cone response and unique hue response as proposed in Fig 5? Arguably not. Consider the case in detail. When a test color of the previous (pre-operation) unique blue’s wavelength and radiance is presented to the observer, s/he finds it no longer unique blue (and may also find it more saturated than previously) but needs to adjust the wavelength a few nanometers for it to appear unique blue. What has happened behind the scenes? Immediately post-operation, with a new and less-yellowish lens than pre-operation, the pre-operation wavelength of unique blue in the *b* chromatic response now stimulates a slightly different (and possibly more saturated) hue from that previously perceived. Subsequently the wavelength of the test color needs adjustment until it matches the observer’s concept or perception of unique blue. The adjustment may be immediate and automatic or over some period of time. The crucial issue is the appearance of the pre-operation wavelength, rather than the relationship between cones and chromatic responses. Clearly, the simple relationship in Fig 5 is not affected or discredited by the need to relearn (or adjust the wavelength of) a unique hue.

## Discussion

The novel concept of cone *primary* and *secondary* outputs satisfactorily solves the mystery of how the S (or other) cone may at any one time exercise the sole or principal influence on one hue (e.g., blue) and a lesser, shared, influence on a second hue (e.g., red), each hue varying independently with changing wavelength or cone excitation. The need for different outputs from a single cone is well known, e.g., in the traditional wiring diagram (Fig 4), and more cogently, from the need to map three cones onto four unique hues. The primary and secondary distinction clarifies and prioritizes relations between cones and perceived hues. In introducing the concept and the terms I am indebted to Ref. 1’s authors for their ingenious experiment and their search for a mechanism able to solve their “incompletely solved mystery.” The concept and the improved schema of cone outputs (Figs 4 and 5) may possibly clarify other problems mentioned in the literature [1–7,10–12] in the transformation from cone responses to chromatic channels. However, the physiological difference between primary and secondary cone outputs remains unknown.

The success of the Fig 5 schema (from Ref. 18) in solving Oh and Sokata’s problem is important in one other respect. By demonstrating the usefulness of this recently published schema and theory [18] in solving an historically well-known and long-standing problem that the traditional model(s) could not solve, it helps establish the new paradigm’s credentials.

## References

[1] Oh S, Sakata K (2015) Do the short-wave cones signal blueness? Color Research Application 40:323–328.

[2] Krauskopf J, Healey DW (1982) Cardinal directions of color space. Vision Research 22:1123–1131. 7147723 10.1016/0042-6989(82)90077-3

[3] Mollon JD, Cavonius CR. The chromatic antagonisms of opponent process theory are not the same as revealed in studies of detection and discrimination In Verriest G, ed, Colour Vision Deficiencies VIII. The Hague: Junk W; 1987 P 473–483.

[4] Abramov I, Gordon J (19994) Color appearance: On seeing red-or yellow, or green, or blue. Ann Rev Psychol 45:451–485. 8135508 10.1146/annurev.ps.45.020194.002315

[5] De Valois RL, De Valois KK, Mahon LE (2000) Contribution of S opponent cells to color appearance. Proc Natl Acad Sci USA 97:512–517. 10618449 10.1073/pnas.97.1.512 PMC26694

[6] Knoblauch K, Shevell SK (2001) Relating cone signals to color appearance: failure of montonicity in yellow/blue. Visual Neurosci 18: 901–906. 10.1017/s0952523801186062 12020080

[7] Burns SA, Elsner AE, Pokorny J, Smith VC (1984) The Abney effect: chromaticity coordinates of unique and other constant hues. Vision Research 24: 479–489. 6740967 10.1016/0042-6989(84)90045-2

[8] Pridmore RW (2007) Effect of purity on hue (Abney effect) in various conditions.

[9] Jameson D, Hurvich L (1955) Some quantitative aspects of an opponent colors theory. I. Chromatic responses and spectral saturation. Journal Optical Society of America 45: 546–552. 10.1364/josa.45.000602 13243163

[10] Ingling CR Jr (1982) The transformation from cone to channel sensitivities. Color Res Appl 7: 191–196.

[11] Ingling CR Jr (1977) The spectral sensitivity of the opponent-color channels. Vision Res 17: 1083–1089. 595418 10.1016/0042-6989(77)90014-1

[12] Drum B (1989) Color scaling of chromatic increments on achromatic backgrounds: Implications for hue signals from individual classes of cones. Color Res. Appl 14: 293–3089.

[13] Hurvich L, Jameson D (1955) Some quantitative aspects of an opponent colors theory. II. Brightness, saturation and hue in normal and dichromatic vision. J Opt Soc Am 45: 602–616. 13243163 10.1364/josa.45.000602

[14] Hurvich LM. Color Vision. Sunderland: Sinauer Associates; 1981.

[15] Jameson D, Hurvich L (1967) Opponent-response functions related to measured cone photopigments. J Opt Soc Am 58: 429–430.

[16] Pridmore RW (2009) Relative wavelength metric for the complete hue cycle: Derivation from complementary wavelengths. Color Res Appl 35: 122–133.

[17] Werner JS, Wooten BR (1979) Opponent chromatic mechanisms: Relation to photopigments and hue naming. J Opt Soc Am 69: 422–434. 458509 10.1364/josa.69.000422

[18] Pridmore RW (2013) Cone photoreceptor sensitivities and unique hue chromatic responses: Correlation and causation imply the physiological basis of unique hues. PLOS ONE 8: e77134 doi: 10.1371/journal.pone.0077134 24204755 10.1371/journal.pone.0077134 PMC3804509

[19] Høyland H, Rausand M. System Reliability Theory: Models and Statistical Methods. New York: John Wiley: 2009.

[20] Nathans J (1999) The evolution and physiology of human color vision: insights from molecular genetic studies of visual pigments. Neuron 24: 299–312. 10571225 10.1016/s0896-6273(00)80845-4

[21] Romeskie MI. Chromatic opponent-response functions of anomalous trichromats. PhD thesis, Brown University, Univ. Microfilms; 1976. 10.1016/0042-6989(78)90007-x 310193

[22] Takahashi S, Ejima Y, Akita M (1985) Effect of light adaptation on the perceptual red-green and yellow-blue opponent-color responses. J. Opt Soc Am A 2: 705–712. 3998885 10.1364/josaa.2.000705

[23] Fuld K (1991) The contribution of chromatic and achromatic valence to spectral saturation. Vision Research 31: 237–246. 2017884 10.1016/0042-6989(91)90114-k

[24] Kulp TD, Fuld K (1995) The prediction of hue and saturation for non-spectral lights. Vision Research 35: 2967–2983. 8533335 10.1016/0042-6989(95)00049-6

[25] Smith VC, Pokorny J (1975) Spectral sensitivity of the foveal photopigments between 400 and 500 nm. Vision Res 15:161–171. 1129973 10.1016/0042-6989(75)90203-5

[26] Judd DB, Wyszecki G. Color in Business, Science, Industry. New York: Wiley & Sons; 1975. Fig. 1.24 (cone spectra as linear transform of CIE color matching functions using Pitt’s primaries).

[27] Estevez O. On the fundamental data-base of normal and dichromatic color vision. PhD thesis, University of Amsterdam, Krips Repro Meppel; 1979

[28] Wyszecki G, Stiles WS. Color Science. New York: John Wiley & Sons; 1982. Table 1(8.2.5) (Konig fundamentals derived by Wyszecki and Stiles)

[29] Wyszecki G, Stiles WS. Color Science. New York: John Wiley & Sons; 1982. Table 2(8.2.5) (Vos and Walraven fundamentals)

[30] Wyszecki G, Stiles WS. Color Science. New York: John Wiley & Sons; 1982. Table 2(7.4.3) (Stiles’ field sensitivities at the fovea)

[31] Dowling JE. The Retina: An Approachable Part of the Brain. Cambridge, Massachusetts: Harvard University Press; 1987.

[32] Vos JJ, Estevez O, Walraven PL (1990) Improved color fundamentals offer a new view on photometric additivity. Vision Res 30: 936–943. 10.1016/0042-6989(90)90059-t 2385931

[33] Stockman A, MacLeod DIA, Johnson NE (1993) Spectral sensitivities of the human cones. J Opt Soc Am A 10: 2491–2521. 10.1364/josaa.10.002491 8301403

[34] Stockman A, Sharpe LT (2000) The middle- and long-wavelength-sensitive cones derived from measurements in observers of known genotype. Vision Res 40:1711–1737. 10814758 10.1016/s0042-6989(00)00021-3

[35] Fairchild MD. Color Appearance Models. 2^nd^ ed. New York John Wiley and Son; 2005.

[36] Pridmore RW (1978) Complementary colors: composition and efficiency in producing various whites. J Opt *Soc Am* 68:1490–1496.

[37] Pridmore RW (1980) Complementary colors: Correction. J Opt Soc Am 70: 248–249.

[38] Wyszecki G, Stiles WS. Color Science. New York: John Wiley & Sons; 1982. Table 3(8.2.6) (“Blue” fundamentals, Bleaching hypothesis and Alternative bleaching hypothesis).

